# Preclinical Immunomodulation by the Probiotic *Bifidobacterium breve* M-16V in Early Life

**DOI:** 10.1371/journal.pone.0166082

**Published:** 2016-11-07

**Authors:** Maria del Mar Rigo-Adrover, Àngels Franch, Margarida Castell, Francisco José Pérez-Cano

**Affiliations:** 1 Departament de Bioquímica i Fisiologia, Facultat de Farmàcia i Ciències de l’Alimentació, University of Barcelona (UB), Barcelona, Spain; 2 Institut de Recerca en Nutrició i Seguretat Alimentària (INSA), Santa Coloma de Gramenet, Barcelona, Spain; University of Palermo, ITALY

## Abstract

This study aimed to investigate the effect of supplementation with the probiotic *Bifidobacterium breve* M-16V on the maturation of the intestinal and circulating immune system during suckling. In order to achieve this purpose, neonatal Lewis rats were supplemented with the probiotic strain from the 6th to the 18th day of life. The animals were weighed during the study, and faecal samples were obtained and evaluated daily. On day 19, rats were euthanized and intestinal wash samples, mesenteric lymph node (MLN) cells, splenocytes and intraepithelial lymphocytes (IEL) were obtained. The probiotic supplementation in early life did not modify the growth curve and did not enhance the systemic immune maturation. However, it increased the proportion of cells bearing TLR4 in the MLN and IEL, and enhanced the percentage of the integrin αEβ7+ and CD62L+ cells in the MLN and that of the integrin αEβ7+ cells in the IEL, suggesting an enhancement of the homing process of naïve T lymphocytes to the MLN, and the retention of activated lymphocytes in the intraepithelial compartment. Interestingly, *B*. *breve* M-16V enhanced the intestinal IgA synthesis. In conclusion, supplementation with the probiotic strain *B*. *breve* M-16V during suckling improves the development of mucosal immunity in early life.

## Introduction

The gut-associated lymphoid tissue (GALT) is a very extensive and complex part of the immune system located in the small intestine and colon. It is organized in inductive sites, which include isolated lymphoid follicles and Peyer’s Patches (PP), where antigens are sampled from the lumen by antigen-presenting cells and the synthesis of IgA is stimulated, and the mesenteric lymph nodes (MLN), where the antigens are presented to naïve lymphocytes which will become activated cells. After moving to the blood, activated lymphocytes migrate again to the intestine where they are diffusely distributed in the *lamina propria* lymphocytes (LPL) and the intraepithelial lymphocytes (IEL) [1].

The gut is colonized by intestinal microbiota, composed of hundreds of bacterial species, which play an important role in the host health. The main functions of the microbiota are protective, metabolic and trophic activities [2,3]. By producing bacteriocins and also by competing for nutrients and attachment sites on the intestinal surface, the microbiota protects the host from pathogen microbes. Furthermore, intestinal bacteria are able to ferment non-digestible carbohydrates and to produce short-chain fatty acids (SCFA), and they are also involved in vitamin synthesis and ion absorption. SCFA (such as acetate, propionate and butyrate) are a source of energy, and also regulate the glucose and lipid metabolism [2,4]. SCFA also have a trophic effect, promoting the proliferation and differentiation of intestinal epithelial cells. Furthermore, butyrate seems to inhibit neoplastic cell proliferation. Finally, bacteria in the lumen interact with the immune system through the pattern recognition receptors, such as the toll-like receptors (TLR), which recognize pathogen-associated molecular patterns [5,6].

The host–microbe interaction is of particular importance in early life. The challenge with antigens is necessary for the maturation of intestine and GALT. The intestinal immune system will acquire the ability to induce tolerance against innocuous dietary, commensal bacterial and self-antigens, and to fight pathogen antigens, in order to maintain intestinal homeostasis and avoid immune-mediated disorders [3,7–9]. Thus, the interaction of microbiota with the intestinal epithelial and immune barriers is involved in the development of oral tolerance and allows microbiota to modulate both innate and adaptive immunity, locally and systemically.

Probiotics are live microorganisms that confer a health benefit on the host when administered in adequate amounts [10]. Different probiotic strains, mainly from *Lactobacillus* and *Bifidobacterium* genera [11], have demonstrated beneficial effects in some disorders, such as diarrhoea, allergy, inflammatory bowel disease (IBD), lactose malabsorption and necrotizing enterocolitis (NEC) in preterm neonates [12]. These results evidence that probiotics, are able to modulate the immune system. Some of the described effects include the modulation of cytokine production by epithelial cells, the increase of mucin secretion, the enhancement of phagocytosis and natural killer (NK) cell activity, the activation of T and NKT cells, the stimulation of IgA production and the reduction of T-cell proliferation [3,5–7,9,13–16]. It has to be taken into account that each effect is strain-dependent, thus a demonstrated action by particular bacteria cannot be attributed to another strain. In this sense, some probiotic strains have shown pro-inflammatory properties (increasingTh1/Th2 ratio), while others have shown anti-inflammatory modulation (decreasingTh1/Th2 ratio). Those stimulating the Th1 cytokine pattern would be useful in preparing the host to fight against infections, and also to ameliorate allergy. But they could be harmful in autoimmune diseases [7], where those promoting anti-inflammatory cytokine patterns would be a better option. There are also probiotic strains stimulating both Th1 and Th2 responses, thus each case needs to be studied individually [7]. The mechanisms of action for this immunomodulation are still not completely known [5,6,13]. Moreover, host microenvironment and enteric commensals, pathogens and vaccines also contributes to diversity of response to identical probiotic strains [17,18].

As explained above, early infancy and childhood are a critical period for immune system programming, and thus they are a window of time of particular interest for the use of probiotics [19,20]. Some authors point out the importance of the establishment of a proper gut microbiota, which depends on several factors, such as the mother’s microbiota, the mother’s diet, the mode of delivery or the use of antibiotics [9,21]. The administration of probiotics to the child during lactation or after weaning, or even to the mother during pregnancy and lactation, has been proposed as a strategy to prevent dysbiosis and its harmful consequences [22,23]. An example of long-term effects is a reduction in the prevalence of attention-deficit hyperactivity disorder and Asperger’s syndrome in 13-year-old boys who were treated with *Lactobacillus rhamnosus* GG (LGG) for the first 6 months of life [24]. Moreover, some effects of the probiotics seem to be more potent in the neonatal period, and even lost later in life, i.e. the modulation of the IgE response [8,9,13]. Hashemi et al. [25] reviewed the recent animal studies of probiotic supplementation in early life and highlighted some effects, such as the improvement of the microbiota composition, the enhancement of the intestinal maturation and immune response, and the reduction in the prevalence of infections.

*Bifidobacterium breve* M-16V is naturally present in infants’ microbiota and has shown immunomodulatory properties. This probiotic seems to ameliorate allergy symptoms, often in combination with prebiotics [26–29], and rotavirus diarrhoea [30]. The aim of the present study was to evaluate the impact of *B*. *breve* M-16V supplementation on some aspects of the immune system development using a neonatal rat as a model. The neonatal rat model is suitable for immunonutrition studies, with substantial scientific evidence and an interesting cost-effective ratio [31]. This model allows the characterization of lymphocyte changes during suckling in several lymphoid compartments [32–35].

## Materials and Methods

### Animals

Four pregnant Lewis rats (G14), obtained from Janvier Labs (Le-Genest-Saint-Isle, France), were housed in individual cages, monitored daily and allowed to deliver naturally. The day of birth was registered as day 1 of life. Litters were unified to 8 pups per lactating dam, with free access to the nipples and rat diet. Dams were fed a commercial diet corresponding to the American Institute of Nutrition 93G formulation and given water *ad libitum*. The animals were housed in controlled conditions of temperature and humidity, in a 12 h:12 h light:dark cycle. Pups were individually identified. They were weighed and monitored daily in order to obtain data regarding the influence of the nutritional intervention on growth and faecal features. This was done after the separation of the pups from their mother, during the handling and before oral administration.

The studies were approved and performed in accordance with the institutional guidelines for the care and use of laboratory animals established by the Ethics Committee for Animal Experimentation of the University of Barcelona and the Catalonian Government (CEEA-UB Ref.493/12, DAAM: 6905).

### Experimental design and dietary supplementation

The first 21 days of a rat’s life corresponds to the lactating 9-month period of humans. The probiotic supplementation began after 1 week of lactation (after the first 1/3 of the period, when strictly only suckling exists) until the end of the study.

The design chosen was that in which the cells from the immune system compartments are still in development (age comprised during the suckling period of the animals) [31–37], but without the interference of breast milk compounds (forcing transition to solid diet on day 17 of life). Moreover, in order to avoid the transitional trapping lymphocytes effects of weaning on immune system [38], last day of experiment was performed on day 19, two days after early weaning.

Suckling rats were distributed into two groups: the reference (REF) and the probiotic (PRO) groups. Each group was composed of 8 pups from two different litters (just half of the pups from each litter were used for this study). The PRO group received *B*. *breve* M-16V (Morinaga Milk Industry Co. Ltd, Tokyo, Japan) suspension at a dose of 4.5x10^8^ UFC/100 g of body weight/day. The REF group was administered with a matched volume of mineral water. Suckling rats were orally administered, as previously described [36], from day 6 to day 18 of age, using low-capacity syringes (Hamilton Bonaduz, Bonaduz Switzerland) adapted to forced alimentation tubes of 25 or 23 calibre and 27 mm of length (ASICO, Westmont, IL, USA).

The animals were weighed and faecal samples were collected daily during the study. At the end of the study intestinal wash samples and spleen and MLN cells and IEL were isolated.

### Faecal specimen collection and evaluation

Faecal sampling was performed once daily by gently pressing and massaging the abdomen. Faecal samples were scored from 1 to 4 (faecal appearance score [FAS]) based on colour, texture and amount as described: normal faeces (1); soft yellow-green faeces (2); totally loose yellow-green faeces (3); high amount of watery faeces (4). Scores ≥ 2 indicate diarrhoeic faeces [37]. Faecal weight for each specimen obtained was also measured.

### Spleen and intestinal samples collection

After ketamine/xylazine injection and exsanguination, the abdomen was opened and MLN, the spleen and the small intestine were collected. The intestine was flushed *in situ* with cold saline solution to remove the content. Thereafter the duodenum was removed. The proximal ¾ portions were used for IEL isolation. The distal ¼ portion of the intestine was used for gut wash for IgA quantification. It was cut in 5 mm pieces, weighed, and incubated with phosphate buffer solution for 10 min in a 37°C shaker. After centrifugation, supernatants were stored at -20°C until analysis [37].

### Isolation and purification of spleen and MLN cells and IEL

Spleen and MLN cells were isolated as previously described [39]. IEL suspensions from the proximal ¾ portion of the intestine were obtained following procedures adapted to neonatal rats and established previously in our laboratory [32]. They were later purified and cell number viability was determined using an automated cell counter after staining dead cells with trypan blue (Countess^TM^, Invitrogen, Madrid, Spain).

### Immunofluorescence staining and flow cytometry analysis

Spleen and MLN cells and IEL (3x10^5^ cells) were stained using immunofluorescence techniques. The mouse anti-rat monoclonal antibody conjugated to fluorescein isothiocyanate, phycoerythrin, peridinin chlorophyll protein or allophycocyanin used here included anti-CD4 (OX-35), anti-CD8α (OX-8), anti-TCRαβ (R73), anti-TCRγδ (V65) and anti-NKR-P1A (10/78), all from BD Pharmingen (San Diego, CA, USA); anti-CD45RA (OX-33) from Caltag (Burlingame, CA, USA); anti-CD8β (3·41) from Serotec (Kidlington, Oxford, UK); anti-TLR4 (76B357.1) from Novus Biologicals (Littleton, CO, USA); anti-αE integrin (OX-62) and anti-CD62L (OX-85) from BioLegend (San Diego, CA, USA).

For cell subset differentiation four different antibody panels were used: Panel 1: CD45RA/TLR4/CD8α/CD4; Panel 2: αE integrin/CD62L/CD8α/CD4; Panel 3: TCRαβ/NK/CD8α/CD4 and Panel 4: CD8α/CD8β/TCRγδ. Fist aim was to dissect main subpopulations. B cells (CR45RA+ cells, which is a B cell marker in rats) were identified with the first panel. With the Panel 3 it could be differentiated NK cells (TCRαβ-NK+) from NKT cells (TCRαβ+NK+), and also TCRαβ+NK- cells, which in combination with the TCRγδ+ cells (obtained from the Panel 4) constituted the total of T cells. Moreover, the CD8+TCRαβ+NK- cells (Panel 3) plus the TCRγδ+ cells (Panel 4) could be considered as Tc cells. From Panel 3 we obtained the proportion of Th cells (CD4+CD8-TCRαβ+NK- cells). Panel 1 was designed for investigating the frequency of TLR4+ cells in each of the subsets defined in this panel (CD45RA+, CD4+ or CD8+ cells). The αE integrin/CD62L pattern was studied by Panel 2 in each of the subsets defined by the combination of the markers used (CD4 and CD8). Gating strategy used in the study can be observed in the S1 Fig.

Results are expressed as proportion of positive cells for a certain marker in each particular subset determined by the combination of other markers with respect to the total lymphocytes gated (i.e. proportion of TLR4+ cells in CD4+CD8- cells).

Staining was developed following procedures described in previous studies [35], and results were analysed using a Gallios^TM^ flow cytometer (Beckman Coulter Inc., Madrid, Spain) in the cytometry service of the Scientific and Technological Centres of the University of Barcelona (CCiT-UB). The obtained data were assessed with FlowJo software (Tree Star Inc., Ashland, OR, USA).

### ELISA for intestinal IgA quantification

Gut wash IgA concentration was determined using a sandwich ELISA technique with the Rat IgA ELISA Quantitation Set (E110-102) from Bethyl Laboratories (Montgomery, TX, USA). Ninety-six well plates (Nunc MaxiSorp, Wiesbaden, Germany) were coated with adequate dilution of the capture antibody. After incubating standard or gut wash samples, the peroxidase conjugated detection antibody was added. Subsequently, substrate solution (3,3′,5,5′-Tetramethylbenzidine plus hydrogen peroxide in dimethyl sulfoxide and 0.05 M phosphate-citrate buffer, pH 5; Sigma-Aldrich, Madrid, Spain) was added and absorbance was measured at 450 nm, after stopping the enzymatic reaction with 2 M H_2_SO_4_, on a microtitre plate photometer (Labsystems, Helsinki, Finland). Data were interpolated by means of Multiskan Ascent v.2.6 software (Thermo Fisher Scientific S.L.U, Barcelona, Spain). Dilutions of rat IgA (Bethyl Laboratoires) ranging from 500 to 15.625 ng/mL were used as a standard in each plate. Data are expressed as ng of IgA per mg of intestinal tissue used for the gut wash.

### Statistical analysis

The PASW Statistics 18 software package (SPSS Inc., Chicago, IL, USA) was used for statistical analysis. Conventional one-way ANOVA test was performed considering the experimental group as the independent variable. When supplementation had a significant effect on the dependent variable, Scheffé’s test was applied. Significant differences were accepted when p<0.05. All the results are expressed as mean ± SEM.

## Results

### Body weight

**Fig 1:**
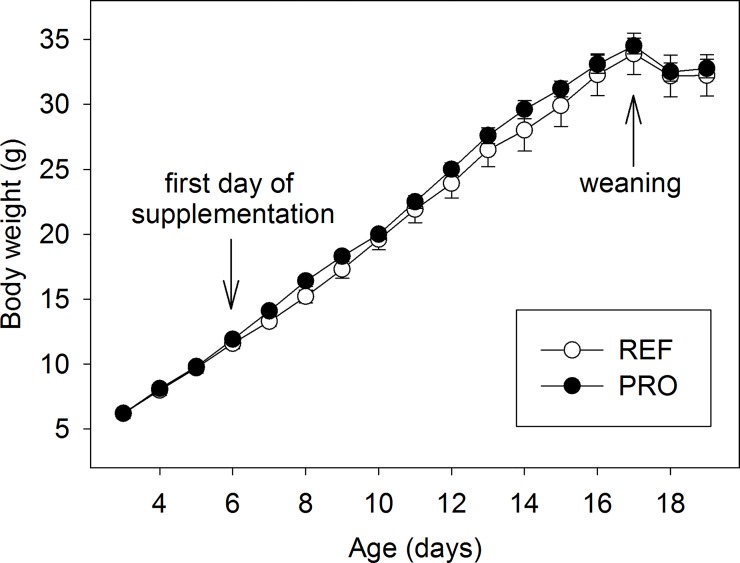
Rats body weight pattern during the study in the reference (REF) and probiotic (PRO) groups. Results are expressed as mean ± SEM (n = 8 animals/group). The first day of nutritional intervention and the weaning day are shown by arrows.

The growth curves of the REF and PRO groups were very similar and without statistical differences throughout the period, as can be seen in Fig 1. Before the PRO intervention (days 3–6), both groups showed identical weight. From day 6 to day 17 there was a slightly higher weight in the PRO group, but still similar (p>0.05). On days 18 and 19, the early weaning effect can be observed in both groups, since there is a slight weight loss during the first days after separation from their dam.

### Faecal consistency

**Fig 2:**
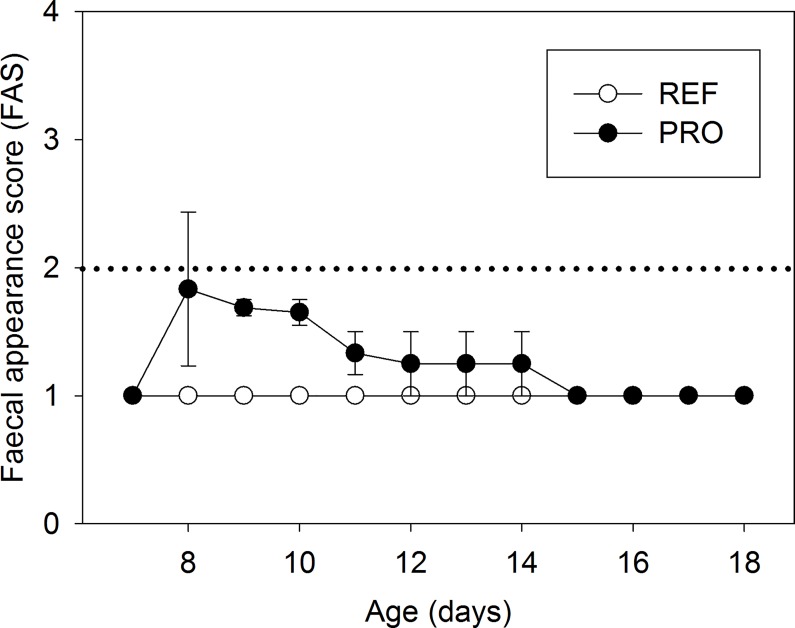
Faecal appearance score (FAS) in the reference (REF) and probiotic (PRO) groups. Faecal samples were scored from 1 to 4 based on colour, texture and amount of stool. Scores of FAS ≥ 2 indicate diarrhoeic faeces. Results are expressed as mean ± SEM (n = 8 animals/group).

The PRO group showed faecal scores slightly higher than 1 (normal faeces) from the second day of supplementation (day 8) until day 14, indicating that the PRO induced soft faeces (Fig 2). However, the faecal weight was 7.60 ± 2.90 mg at the beginning of the supplementation and 10.44 ± 1.83 mg at the end for both groups, without significant differences between groups.

### Phenotype of spleen and MLN cells and IEL

On the last day of the study (day 19) spleen and MLN cells, as well as IEL, were isolated in order to evaluate the influence of the PRO on the lymphocytes. A sufficient number of viable cells from all tissues were obtained (with a viable cell number range of 1.7–4.6x10^7^ and a viability of 73–98% for spleen cells; 1.7x10^5^–1.9x10^6^ and 72–91% for MLN cells; 4.5x10^5^–1.1x10^6^ and 76–87% for IEL). No differences were observed between the REF and PRO groups.

#### Main lymphocyte subset proportions

The phenotype of lymphocyte subsets present in systemic (spleen) and intestinal compartments (MLN and IEL) was studied. The percentage of the main subsets present in each compartment is summarized in Table 1.

**Table 1:** Main lymphocyte subsets in the spleen, mesenteric lymph nodes (MLN) and intraepithelial lymphocytes (IEL) of the reference (REF) and probiotic (PRO) groups.

				Spleen		MLN		IEL	
				REF	PRO	REF	PRO	REF	PRO
**B cells** (CD45RA+)				33.00 ± 1.82	33.60 ± 1.41	12.15 ± 1.57	13.99 ± 0.72	-	-
**T cells** (TCRαβ+NK- and TCRγδ+)				25.85 ± 0.57	22.32 ± 0.62*	74.78 ± 1.56	70.37 ± 1.69	25.85 ± 0.57	25.85 ± 0.57
**Th cells** (CD4+CD8-TCRαβ+)				15.95 ± 0.40	13.55 ± 0.60*	55.43 ± 1.32	52.00 ± 1.45	2.55 ± 0.20	1.58 ± 0.11*
	CD4+CD8-			26.11 ± 0.54	23.09 ± 0.49*	59.91 ± 1.58	56.96 ± 1.37	3.35 ± 0.16	2.49 ± 0.14*
	CD4+CD8- TCRαβ-			10.16 ± 0.53	9.54 ± 0.52	4.48 ± 0.40	4.96 ± 0.40	0.80 ± 0.29	0.91 ± 0.17
**Tc cells** (CD8+TCRαβ+NK- and TCRγδ+)				9.90 ± 0.20	8.77 ± 0.23*	19.35 ± 0.52	18.37 ± 0.62	20.28 ± 0.21	19.33 ± 0.44
	**TCRαβ+** (CD8+ TCRαβ+NK-)			8.17 ± 0.24	7.22 ± 0.16*	17.10 ± 0.55	16.21 ± 0.54	8.17 ± 0.60	7.98 ± 0.24
	**TCRγδ+**			1.73 ± 0.06	1.55 ± 0.09	2.26 ± 0.03	2.16 ± 0.12	12.11 ± 0.77	11.35 ± 0.34
		CD8-TCRγδ+		0.35 ± 0.03	0.29 ± 0.01	0.38 ± 0.02	0.34 ± 0.02	0.66 ± 0.09	0.57 ± 0.06
		CD8+TCRγδ+		1.38 ± 0.04	1.26 ± 0.09	1.88 ± 0.03	1.82 ± 0.11	11.45 ± 0.73	10.78 ± 0.35
			CD8αα+TCRγδ+	0.16 ± 0.02	0.17 ± 0.01	0.21 ± 0.02	0.19 ± 0.04	8.04 ± 0.80	7.89 ± 0.27
			CD8αβ+TCRγδ+	1.21 ± 0.04	1.10 ± 0.08	1.67 ± 0.03	1.62 ± 0.09	3.41 ± 0.16	2.89 ± 0.14*
**NKT cells** (TCRαβ+NK+)				2.91 ± 0.06	2.61 ± 0.23	1.81 ± 0.08	1.87 ± 0.16	21.00 ± 0.57	21.06 ± 0.65
**NK cells** (TCRαβ-NK+)				7.18 ± 0.19	6.02 ± 0.73	1.18 ± 0.17	1.49 ± 0.15	36.18 ± 0.99	36.89 ± 0.85
	CD8+/TCRαβ-NK+			3.55 ± 0.13	3.23 ± 0.38	0.19 ± 0.02	0.27 ± 0.02*	22.02 ± 0.85	21.29 ± 0.36
**CD8αα+ cells**				2.49 ± 0.12	2.31 ± 0.24	1.09 ± 0.06	1.27 ± 0.18	21.95 ± 1.49	21.81 ± 0.63
**CD8αβ+ cells**				10.70 ± 0.11	9.58 ± 0.43*	18.90 ± 0.47	17.49 ± 0.56	21.50 ± 0.61	21.27 ± 0.81
**Ratio CD8αα/CD8αβ**				0.23 ± 0.01	0.24 ± 0.01	0.06 ± 0.00	0.07 ± 0.01	1.02 ± 0.04	1.03 ± 0.03

Results are expressed as percentage of the total lymphocytes (mean ± SEM, n = 8).

* p<0.05 *vs*. REF.

Among the spleen cells, the PRO nutritional intervention did not affect B cell proportion but was able to significantly reduce the proportion of both T helper (Th) and cytotoxic T (Tc) cells (p<0.05). However, the administration of the PRO did not influence any of the TCRγδ+ subtypes, nor the NK or NKT cell proportion. In addition, when the percentage of cells bearing CD8 co-receptor structure was studied, although a decrease of CD8αβ+ cell proportion was observed due to PRO supplementation it did not modify the CD8αα+/CD8αβ+ ratio.

Regarding the MLN, the main subsets proportion (B, Th, Tc, NKT and NK) were not affected by PRO diet, although an increase in the CD8+ NK population was observed (p<0.05). Most of the lymphocytes in the MLN with the CD8 co-receptor were CD8αβ+, with no effect caused by the PRO supplementation.

On the other hand, when the IEL composition was studied, it was observed that the Th cell proportion was reduced significantly by the nutritional intervention (p<0.05). Although the Tc cell proportion was not modified, the CD8αβ+TCRγδ+ subset percentage was significantly reduced (p<0.05). Finally, there was a similar proportion of both CD8αα and CD8αβ IEL in both groups without any effect from the PRO supplementation. This CD8αα/CD8αβ ratio was the highest among the three compartments studied.

#### TLR4 cell surface expression

**Fig 3:**
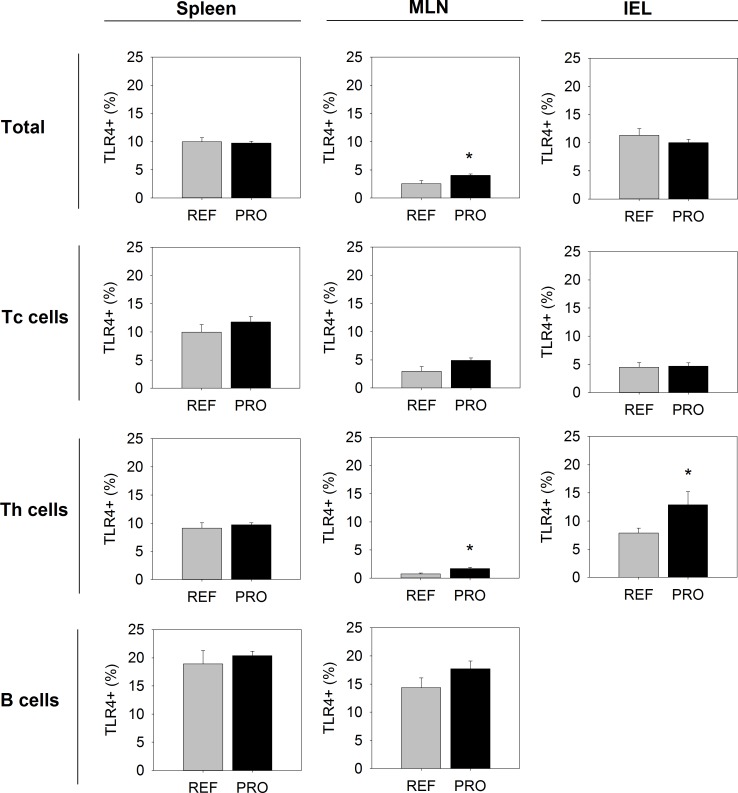
Proportion of TLR4+ cells in the main lymphocyte subsets from the three compartments studied (spleen, mesenteric lymph nodes and intraepithelial lymphocytes) of the reference (REF) and probiotic (PRO) groups. Results are expressed as percentage of positive cells in the indicated subset (mean ± SEM, n = 8 animals/group). Statistical differences: *p<0.05 *vs*. REF.

The ability of developing lymphocytes to interact with bacteria was investigated by studying the presence of the TLR4 in the main cell subsets in spleen, MLN and IEL (Fig 3). The proportion of spleen B cells expressing TLR4 (~20%) was higher than that in T cells (~10%). No differences in TLR4+ cells were observed between the REF and the PRO groups in the spleen, when explored either in whole lymphocytes or in each cell subset (Tc, Th or B).

In the MLN, the proportion of B cells presenting TLR4 (~15%) was also higher than that in T cells (~5%). In this compartment, the proportion of cells bearing TLR4 was higher in the lymphocytes of the PRO group compared to the REF group. The percentage of TLR4+ cells in the PRO group was higher in Tc, Th and B lymphocytes than that in the REF group, although the difference did only achieve statistical significance for the Th cells (p<0.05).

Gut IEL had similar proportions of cells expressing TLR4 in both the REF and PRO groups, with the exception of the percentage of TLR4+ Th cells, which was higher in the PRO group (p<0.05).

#### Cell surface expression of αE integrin and CD62L molecules

**Fig 4:**
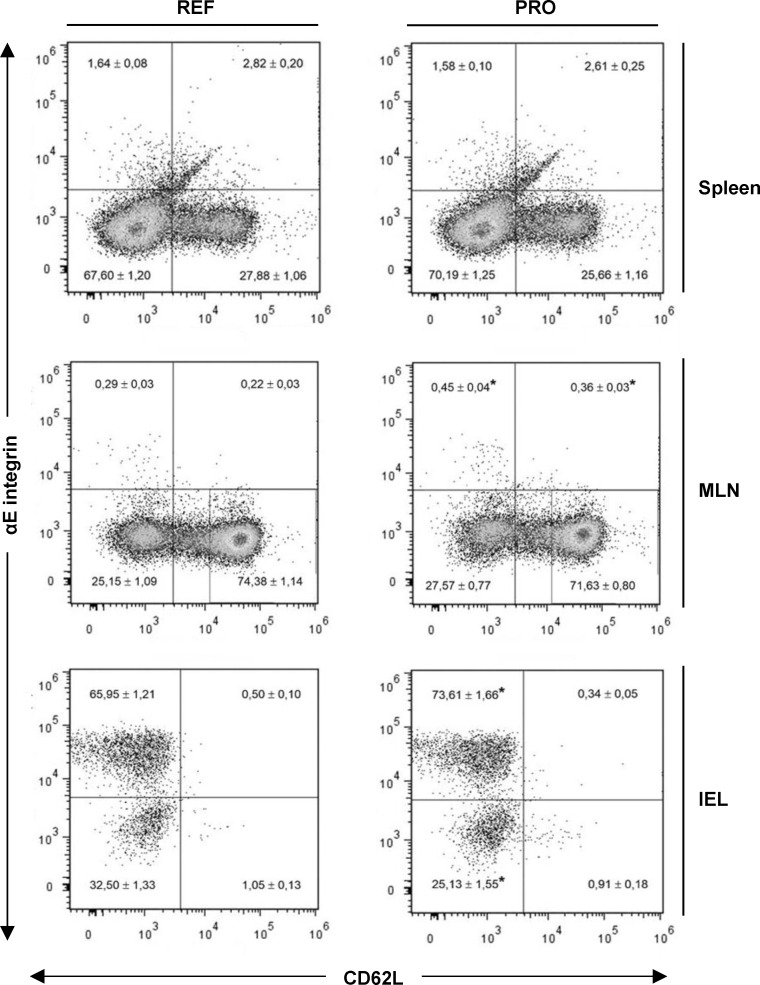
αE integrin/CD62L molecular pattern in the total lymphocytes of the spleen, mesenteric lymph nodes (MLN) and intraepithelial lymphocytes (IEL). Representative histograms for the reference (REF) and probiotic (PRO) groups are shown. In each quadrant the mean ± SEM (n = 8 animals/group) is included. Statistical differences: *p<0.05 *vs*. REF.

The lymphocytes’ commitment to the mucosal compartment was studied by means of the proportion of cells expressing two adhesion molecules of importance in the intestinal homing (Fig 4). As expected, the αE integrin/CD62L expression pattern was different among splenocytes, MLN cells and IEL, with CD62L being highly expressed in organized tissues (spleen and MLN) with respect to IEL, and an inverse behaviour found for αE integrin (Fig 4). Focusing on the spleen, no changes were found in the proportion of cells bearing αE integrin and/or CD62L in the REF and PRO groups. The αE integrin/CD62L pattern was also studied in particular subsets based on the CD4/CD8 cell distribution. In this sense, the proportion of αE+CD62L-, αE+CD62L+, αE-CD62L+ and the αE-CD62L- cells was studied in the CD4+CD8-, CD4+CD8+, CD4-CD8+ and the CD4-CD8- subsets and no statistical differences were found (Table 2).

**Table 2:** Expression of the integrin αE and the selectin CD62L in CD4+, CD8+ and CD4- CD8- patterns in spleen, mesenteric lymph node (MLN) cells and intraepithelial lymphocytes (IEL) of the reference (REF) and probiotic (PRO) groups.

		αE+CD62L-		αE+CD62L+		αE-CD62L+		αE-CD62L-	
		REF	PRO	REF	PRO	REF	PRO	REF	PRO
**Spleen**	**CD4+CD8-**	1.83 ± 0.09	2.01 ± 0.16	0.90 ± 0.12	0.71 ± 0.08	42.23 ± 0.91	41.54 ± 2.34	55.08 ± 0.73	55.73 ± 2.27
	**CD4+CD8+**	11.56 ± 1.08	12.01 ± 0.52	44.63 ± 1.47	45.67 ± 1.65	16.40 ± 1.38	14.06 ± 1.15	27.40 ± 1.81	28.26 ± 1.27
	**CD4-CD8+**	1.23 ± 0.12	1.41 ± 0.19	2.42 ± 0.48	2.28 ± 0.21	57.58 ± 2.17	52.66 ± 1.62	38.78 ± 1.89	43.66 ± 1.58
	**CD4-CD8-**	0.87 ± 0.07	0.78 ± 0.03	0.38 ± 0.06	0.27 ± 0.02	17.83 ± 1.89	17.30 ± 0.97	80.95 ± 2.01	81.61 ± 1.00
**MLN**	**CD4+CD8-**	0.18 ± 0.05	0.24 ± 0.03	0.20 ± 0.05	0.27 ± 0.03	78.73 ± 1.34	77.85 ± 0.60	20.88 ± 1.28	21.65 ± 0.56
	**CD4+CD8+**	0.24 ± 0.09	1.35 ± 0.35*	1.07 ± 0.23	1.90 ± 0.41	84.63 ± 2.06	81.42 ± 1.18	14.08 ± 2.05	15.35 ± 1.04
	**CD4-CD8+**	0.47 ± 0.06	0.74 ± 0.11	0.40 ± 0.09	0.51 ± 0.04	77.85 ± 1.37	78.52 ± 0.75	21.30 ± 1.33	20.23 ± 0.63
	**CD4-CD8-**	0.60 ± 0.04	0.86 ± 0.05*	0.29 ± 0.04	0.47 ± 0.06*	59.65 ± 1.79	52.58 ± 2.16*	39.43 ± 1.71	46.08 ± 2.19*
**IEL**	**CD4+CD8-**	2.21 ± 1.06	6.31 ± 0.73*	0.46 ± 0.30	0.30 ± 0.20	9.00 ± 2.86	8.09 ± 1.91	88.35 ± 2.14	85.30 ± 2.02
	**CD4+CD8+**	29.09 ± 10.05	33.21 ± 9.21	9.46 ± 5.90	13.81 ± 4.62	11.90 ± 4.31	8.96 ± 2.53	49.55 ± 8.49	44.01 ± 5.03
	**CD4-CD8+**	93.58 ± 0.57	95.24 ± 0.54	0.52 ± 0.09	0.37 ± 0.04	1.36 ± 0.25	1.00 ± 0.21	4.54 ± 0.53	3.39 ± 0.51
	**CD4-CD8-**	50.20 ± 2.67	60.17 ± 1.80*	0.37 ± 0.07	0.27 ± 0.06	0.38 ± 0.05	0.36 ± 0.04	49.03 ± 2.73	39.19 ± 1.76*

* p<0.05 *vs*. REF.

In the MLN, cells expressing CD62L were ~ 3-fold more abundant than in the spleen. Integrin αE+ cell proportion was very low in comparison with that in the spleen, and the percentage of cells expressing αE integrin, either with or without CD62L co-expression, was higher in the PRO group (0.81 ± 0.06) than in the REF group (0.51 ± 0.06) (p<0.05). When the expression of these markers was analysed in particular subsets (CD4+, CD8+ and CD4- CD8-), it was observed that the increase in such a pattern due to the PRO intervention was found in all of them (Table 2).

CD62L was almost completely absent on the surface of the IEL but more than 65% expressed αE integrin. In the PRO group, the integrin αE+ cell proportion (73.95 ± 1.66) was 8% higher than that in the REF group (66.45 ± 1.24) (p<0.05). Again, the increase of integrin αE+ IEL in the PRO group was found in all CD4+, CD8+ and CD4- CD8- subsets (p<0.05) and therefore it could not be ascribed to one of these cell types (Table 2).

### Intestinal IgA

**Fig 5:**
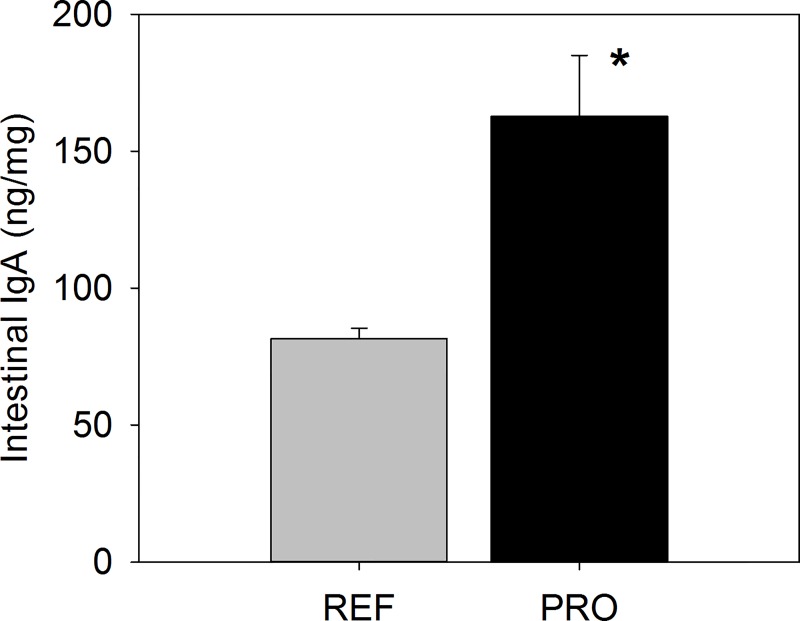
IgA concentration in intestinal washes of 19-day-old rats from the reference (REF) and probiotic (PRO) groups. Results are expressed as ng of IgA/mg of tissue (mean ± SEM, n = 8 animals/group). Statistical differences: *p<0.05 *vs*. REF.

Intestinal IgA was quantified in gut washes from 19-day-old rats. The administration of the PRO only during 2 weeks of the suckling period (starting after the first week of life) was enough to show an enhancing effect on IgA production (Fig 5). In fact, the PRO dietary intervention increased the intestinal IgA concentration 2-fold (p<0.05).

## Discussion

The current study shows that supplementation with the probiotic strain *B*. *breve* M-16V during the rat suckling period influences the lymphocyte composition of both intestinal and systemic immune compartments, modifies the proportion of cells expressing molecules involved in the interaction with intestinal bacteria, and also enhances the intestinal antibody synthesis.

In our study, the PRO administration induced no harmful effects because it did not affect the growth curve. Interestingly, a change in the faecal consistency caused by the PRO was found. This effect has already been described for some molecules used as probiotic substrate [40–42], and it is actually beneficial to make faeces more similar to those of breast-fed infants and to reduce constipation.

During suckling, rats undergo phenotypical changes in lymphoid tissues that are a reflex of the immune system’s maturation in this period. Thus, immature rat spleen mainly contains B cells, T, NK and NKT cell proportions that are lower than those in adult rats [32]. As the PRO supplementation did not produce an increase but rather a decrease in CD4+ and CD8+ cells, it seems that, apparently, this nutritional intervention does not enhance the systemic immune maturation. However, when considering the MLN, a secondary lymphoid organ located at intestinal level and inducer of immune response in this compartment, the PRO supplementation was able to enhance the proportion of CD8+ NK cells. These cells, although were found in a low proportion in MLN, are key in the innate immunity [35] and therefore the current results show that the PRO administration increased this defensive barrier.

With regard to the IEL, these cells are those most in contact with the intestinal lumen and therefore interventional diets can have major impact on them. This compartment does not contain B cells, and during suckling there is a progressive increase in the proportion of CD8+ IEL co-expressing TCRαβ, whereas the percentage of CD8+ cells expressing the NK receptor decreases [32]. In addition, there is an increase in the proportion of CD4+ cells and TCRγδ+ cells during the second half of the suckling period [32]. The PRO administration during suckling did not produce any significant change in the proportion of CD8+ TCRαβ+ IEL or CD8+ NK cells, but lowered the percentage of CD4+ and the proportion of the particular CD8αβ+ TCRγδ+ IEL, which are the least abundant in TCRγδ+ cells in the intraepithelial compartment. These results agree with Gebert et al. [43] who found a decrease in the CD4+ jejunum population of neonatal pigs after *Lactobacillus brevis* supplementation. Some authors also describe a reduction of CD4+ T cells in the mucosa obtained from patients with Crohn’s disease when co-cultured with *Lactobacillus casei* and *Lactobacillus bulgaricus*, but no changes were observed in non-inflamed mucosa [44,45].

In all the three compartments studied, the presence of TLR4 in the main cell subsets was assessed. The TLR4 receptor recognizes the bacterial lipopolysaccharide. It participates in the innate immune response against pathogens, but also in the proliferation, differentiation and development of lymphocytes [46]. TLR4 is essential for the maintenance of the intestinal homeostasis and barrier function [5,8]. The proportion of splenocytes bearing TLR4 was not changed by the nutritional intervention but it was increased due to the PRO supplementation in the CD4+ subset in the MLN and IEL. Therefore, it can be suggested that the PRO can prepare the intestinal immune system for a better response against infections increasing bacteria–host interaction These results agree with those reported after the administration of *Lactobacillus reuteri*, *L*. *casei* and *L*. *rhamnosus* in healthy mice [14,47] and by LGG in healthy rats [48]. However, our findings did not match when TLR4 expression was studied in some pathologies. In this context, TLR4 expression was decreased by *L*. *reuteri* in the intestine of rats with NEC [49], by *L*. *rhamnosus* and *Lactobacillus acidophilus* in mice with alcoholic liver disease [50], and also by LGG and other *Lactobacillus* and *Bifidobacterium* in the colon of rats with colitis [48,51].

The circulation is important in leading naïve lymphocytes to find their specific antigen, as well as for permitting effector or memory lymphocytes to reach infected or inflamed tissue. Intestinal homing depends on the expression of specific surface molecules [52]. The homing of naïve T lymphocytes in the PP or MLN through the high endothelial venules (HEV) requires the interaction between the CD62L selectin on the lymphocyte surface and the peripheral node addressin on the HEV [53]. Moreover, α4β7 integrin is also important to bind to epithelial cells from the small intestine, and to allow the retention of the IEL [54,55]. Interestingly, the CD62L and αEβ7 integrin pattern in the MLN cells and the IEL of suckling rats changed after the administration of *B*. *breve* M-16V, which induced a higher percentage of integrin αEβ7+ cells both in the MLN and the intraepithelial compartment. These results seem to indicate an enhancement of the homing process of naïve T lymphocytes to the MLN, and the retention of activated lymphocytes in the IEL compartment [56]. Other studies have evidenced the capacity of probiotics to modulate homing markers. *Lactobacillus plantarum* and its excreted peptide STp have shown stimulation of skin-homing markers *in vitro*, such as cutaneous lymphocyte-associated antigen (CLA), and a decrease in MadCAM-1 and α4β7 integrin expression in colonic mucosa in a mouse model of colitis [57–59]. These effects can be useful to attenuate the intestinal inflammation in IBD. On the other hand, *L*. *casei* Shirota failed in modulating the homing markers on the stimulated T cells by the dendritic cells of patients with ulcerative colitis, but it did increase the CLA skin-homing marker and β7 gut-homing marker on stimulated T cells by human dendritic cells from healthy controls [60].

On the other hand, and importantly, the PRO administration for 13 days during the suckling period was able to enhance the intestinal IgA synthesis. As expected, the levels of IgA in the intestinal wash samples of the REF animals were very low [20,33], but *B*. *breve* M-16V doubled the intestinal IgA concentration. Since intestinal IgA is a very reliable marker of the mucosal defence against pathogens and has been clearly described as a useful tool in immunonutrition studies [5], this result indicates the immunomodulatory potential of this probiotic strain. Other studies have reported stimulation of IgA production due to probiotics, such as LGG [61], *Lactobacillus gasseri* [9], *L*. *casei*, *L*. *reuteri*, *Bacillus cereus* var. Toyoi, *Bifidobacterium bifidum* and *Lactobacillus kefiri* in mice [23,25,62] or a formula containing *Bifidobacterium lactis* Bb-12 in healthy infants [63].

Finally, it must be considered that in the design of the current study, pups were supplemented from day 6 until the end of the study, and were weaned at day 17, a few days after rats begin to chew solid food. The effects found here demonstrate that supplementation with the specific strain *B*. *breve* M-16V in this short period is enough to show effect, which is in agreement with a study with mice in which just 2 days of supplementation with *Lactobacillus delbrueckii* subsp. *bulgaricus* or *L*. *acidophilus*, and 7 days with *L*.*casei* were necessary to find an increase of IgA in the LP of the small intestine [5]. However, we cannot discount the possibility that a new study design with an earlier and longer administration period would show more significant results.

In summary, the results shown here allow the conclusion to be drawn that the probiotic intervention during suckling seems to have a low, if any, immune-enhancing effect on the systemic compartment (spleen), but *B*. *breve* M16-V administration enhances the ability to respond against pathogens in both the intestinal inductor (MLN) and effector (IEL) sites and it promotes the intestinal recruitment and retention of the cells in the epithelial compartment. In addition, this probiotic strengthens the humoral intestinal immune response. Thus, supplementation with the probiotic strain *B*. *breve* M-16V during suckling may be helpful in the development of mucosal immunity in early life.

## Supporting Information

S1 Fig Example of the gating strategy using a mesenteric lymph node lymphocytes’ sample. **A)** In Panel 1, B cells (CD45RA+) were identified. The frequency of TLR4+ cells in the B cell subset was investigated, as well as in CD8+CD4- and CD8-CD4+ subsets. TLR4+ cells were also quantified in the total gated lymphocytes (not shown). **B)** In Panel 2, the αE integrin/CD62L pattern was studied in each of the subsets defined by the combination of the markers CD4 and CD8. **C)** In Panel 3, it could be differentiated NK (NK+TCRαβ-) cells from NKT cells (NK+TCRαβ+), and also NK-TCRαβ+ cells, which in combination with TCRγδ+ cells (obtained from the Panel 4) constituted the total of T cells. Moreover, the CD8-CD4+ cells in the NK-TCRαβ+ subset could be considered as Th cells, and the CD8+CD4- cells in this subset plus the TCRγδ+ cells (Panel 4) could be considered as Tc cells. The proportion of CD8+ cells in the NK+TCRαβ- subset (NK cells) was also studied. **D)** In Panel 4, the proportion of CD8αα and CD8αβ cells were studied in the CD8+ cells subset and also in the TCRγδ+ cells subset.
(DOCX)

## References

[1] Agace WW. T-cell recruitment to the intestinal mucosa. Trends Immunol. 2008;29: 514–522. 10.1016/j.it.2008.08.003 18838302

[2] Guarner F, Malagelada JR. Gut flora in health and disease. Lancet. 2003;361: 512–519. 10.1016/S0140-6736(03)12489-0 12583961

[3] Aureli P, Capurso L, Castellazzi AM, Clerici M, Giovannini M, Morelli L, et al Probiotics and health: An evidence-based review. Pharmacol Res. 2011;63: 366–376. 10.1016/j.phrs.2011.02.006 21349334

[4] Macfarlane GT, Macfarlane S. Fermentation in the human large intestine: its physiologic consequences and the potential contribution of prebiotics. J Clin Gastroenterol. 2011;45l: S120–S127. 10.1097/MCG.0b013e31822fecfe 21992950

[5] Maldonado Galdeano C, De Moreno De Leblanc A, Vinderola G, Bibas Bonet ME, Perdigón G. Proposed model: Mechanisms of immunomodulation induced by probiotic bacteria. Clin Vaccine Immunol. 2007;14: 485–492. 10.1128/CVI.00406-06 17360855 PMC1865623

[6] Bermudez-Brito M, Plaza-Díaz J, Muñoz-Quezada S, Gómez-Llorente C, Gil A. Probiotic mechanisms of action. Ann Nutr Metab. 2012;61: 160–174. 10.1159/000342079 23037511

[7] Delcenserie V, Martel D, Lamoureux M, Amiot J, Boutin Y, Roy D. Immunomodulatory effects of probiotics in the intestinal tract. Curr Issues Mol Biol. 2008;10: 37–54. 18525105

[8] Kaplan JL, Shi HN, Walker WA. The role of microbes in developmental immunologic programming. Pediatr Res. 2011;69: 465–472. 10.1203/PDR.0b013e318217638a 21364495

[9] Frei R, Akdis M, O’Mahony L. Prebiotics, probiotics, synbiotics, and the immune system. Curr Opin Gastroenterol. 2015;31: 153–158. 10.1097/MOG.0000000000000151 25594887

[10] FAO, WHO. Probiotics in food: Health and nutritional properties and guidelines for evaluation. 2006. In: www.fao.org [Internet]. Available: www.fao.org. Accessed 2016 Feb 21.

[11] Vrese M, Schrezenmeir J. Probiotics, prebiotics, and synbiotics. In: Stahl U, Donalies UEB, Nevoigt E, Scheper T, editors. Advances in Biochemical Engineering/Biotechnology: Food biotechnology. 2008. pp. 1–66. 10.1007/10_2008_097 10.1007/10_2008_097 18461293

[12] Guarner F, Khan AG, Garisch J, Eliakim R, Gangl A, Thomson A, et al World gastroenterology organisation global guidelines: probiotics and prebiotics october 2011. J Clin Gastroenterol. 2012;46: 468–481. 10.1097/MCG.0b013e3182549092 22688142

[13] Bodera P, Chcialowski A. Immunomodulatory effect of probiotic bacteria. Recent Pat Inflamm Allergy Drug Discov. 2009;3: 58–64. 19149747 10.2174/187221309787158461

[14] Kotzamanidis C, Kourelis A, Litopoulou-Tzanetaki E, Tzanetakis N, Yiangou M. Evaluation of adhesion capacity, cell surface traits and immunomodulatory activity of presumptive probiotic Lactobacillus strains. Int J Food Microbiol. 2010;140: 154–163. 10.1016/j.ijfoodmicro.2010.04.004 20452079

[15] Pérez-Cano FJ, Dong H, Yaqoob P. In vitro immunomodulatory activity of Lactobacillus fermentum CECT5716 and Lactobacillus salivarius CECT5713: two probiotic strains isolated from human breast milk. Immunobiology. 2010;215: 996–1004. 10.1016/j.imbio.2010.01.004 20219262

[16] Smelt MJ, de Haan BJ, Bron PA, van Swam I, Meijerink M, Wells JM, et al Probiotics can generate FoxP3 T-cell responses in the small intestine and simultaneously inducing CD4 and CD8 T cell activation in the large intestine. PLoS One. 2013;8: e68952–e68952. 10.1371/journal.pone.0068952 23861953 PMC3701681

[17] Yan F, Polk DB. Probiotics and immune health. Curr Opin Gastroenterol. 2011;27: 496–501. 10.1097/MOG.0b013e32834baa4d 21897224 PMC4006993

[18] Serazin AC, Shackelton LA, Wilson C, Bhan MK. Improving the performance of enteric vaccines in the developing world. 2010;11: 769–773. 10.1038/ni0910-769 20720580

[19] Gueimonde M, Kalliomäki M, Isolauri E, Salminen S. Probiotic intervention in neonates-will permanent colonization ensue? J Pediatr Gastroenterol Nutr. 2006;42: 604–606. 10.1097/01.mpg.0000221897.45910.d3 16707993

[20] Martin R, Nauta AJ, Ben Amor K, Knippels LMJ, Knol J, Garssen J. Early life: Gut microbiota and immune development in infancy. Benef Microbes. 2010;1: 367–382. 10.3920/BM2010.0027 21831776

[21] Bertelsen RJ, Jensen ET. Use of probiotics and prebiotics in infant formulas. Best Pract Res Clin Gastroenterol. 2016;30: 39–48. 10.1016/j.bpg.2016.01.001 27048895

[22] Escribano E, Moles L, De Andres J, Jimenez E, Espinosa-Martos I, Rodriguez JM, et al Administration of Bifidobacterium breve PS12929 and Lactobacillus salivarius PS12934, two strains isolated from human milk, to very low and extremely low birth weight preterm infants: A pilot study. Arch Dis Child. 2014;Conference: 5th Congress of the European Academy of Paediatric. 10.1136/archdischild-2014-307384.1249 PMC4352454 25759843

[23] Carasi P, Racedo SM, Jacquot C, Romanin DE, Serradell MA, Urdaci MC. Impact of kefir derived Lactobacillus kefiri on the mucosal immune response and gut microbiota. J Immunol Res. 2015;2015: 361604 10.1155/2015/361604 25811034 PMC4355334

[24] Pärtty A, Kalliomäki M, Wacklin P, Salminen S, Isolauri E. A possible link between early probiotic intervention and the risk of neuropsychiatric disorders later in childhood—a randomized trial. Pediatr Res. 2015;77: 823–828. 10.1038/pr.2015.51 25760553

[25] Hashemi A, Villa CR, Comelli EM. Probiotics in early life: a preventative and treatment approach. Food Funct. 2016;In press. 10.1039/C5FO01148E 26979945

[26] Hougee S, Vriesema AJM, Wijering SC, Knippels LMJ, Folkerts G, Nijkamp FP, et al Oral treatment with probiotics reduces allergic symptoms in ovalbumin-sensitized mice: A bacterial strain comparative study. Int Arch Allergy Immunol. 2010;151: 107–117. 10.1159/000236000 19752564

[27] Van De Pol MA, Lutter R, Smids BS, Weersink EJM, Van Der Zee JS. Synbiotics reduce allergen-induced T-helper 2 response and improve peak expiratory flow in allergic asthmatics. Allergy Eur J Allergy Clin Immunol. 2011;66: 39–47. 10.1111/j.1398-9995.2010.02454.x 20716319

[28] De Kivit S, Saeland E, Kraneveld AD, Van De Kant HJG, Schouten B, Van Esch BCAM, et al Galectin-9 induced by dietary synbiotics is involved in suppression of allergic symptoms in mice and humans. Allergy Eur J Allergy Clin Immunol. 2012;67: 343–352. 10.1111/j.1398-9995.2011.02771.x 22229637

[29] Verheijden KAT, Willemsen LEM, Braber S, Leusink-Muis T, Jeurink PV, Garssen J, et al The development of allergic inflammation in a murine house dust mite asthma model is suppressed by synbiotic mixtures of non-digestible oligosaccharides and Bifidobacterium breve M-16V. Eur J Nutr. 2016;55: 1141–1151. 10.1007/s00394-015-0928-8 26003185 PMC4819948

[30] Rigo-Adrover M, Saldaña-Ruíz S, van Limpt K, Knipping K, Garssen J, Knol J, et al A combination of scGOS/lcFOS with Bifidobacterium breve M-16V protects suckling rats from rotavirus gastroenteritis. Eur J Nutr. 2016;In press. 10.1007/s00394-016-1213-1 27112962

[31] Pérez-Cano FJ, Franch A, Castellote C, Castell M. The suckling rat as a model for immunonutrition studies in early life. Clin Dev Immunol. 2012;2012: 537310 10.1155/2012/537310 22899949 PMC3415261

[32] Pérez-Cano FJ, Castellote C, González-Castro AM, Pelegrí C, Castell M, Franch A. Developmental changes in intraepithelial T lymphocytes and NK cells in the small intestine of neonatal rats. Pediatr Res. 2005;58: 885–891. 10.1203/01.pdr.0000182187.88505.49 16257927

[33] Pérez-Cano FJ, Castellote C, Marín-Gallén S, Franch A, Castell M. Neonatal immunoglobulin secretion and lymphocyte phenotype in rat small intestine lamina propria. Pediatr Res. 2005;58: 164–169. 10.1203/01.PDR.0000156367.60769.36 15774849

[34] Pérez-Cano FJ, Castellote C, Marín-Gallén S, González-Castro A, Franch A, Castell M. Phenotypic and functional characteristics of rat spleen lymphocytes during suckling. Dev Comp Immunol. 2007;31: 1264–1277. 10.1016/j.dci.2007.03.004 17459475

[35] Marín-Gallén S, Pérez-Cano FJ, Castell M, Castellote C, Franch A. Intestinal intraepithelial NK and NKT cell ontogeny in Lewis rats. Dev Comp Immunol. 2008;32: 1405–1408. 10.1016/j.dci.2008.06.011 18638501

[36] Pérez-Cano FJ, Marín-Gallén S, Castell M, Rodríguez-Palmero M, Rivero M, Franch A, et al Bovine whey protein concentrate supplementation modulates maturation of immune system in suckling rats. Br J Nutr. 2007;98: S80–S84. 10.1017/S0007114507838074 17922966

[37] Pérez-Cano FJ, Castell M, Castellote C, Franch A. Characterization of clinical and immune response in a rotavirus diarrhea model in suckling Lewis rats. Pediatr Res. 2007;62: 658–663. 10.1203/PDR.0b013e318159a273 17957154

[38] Kick AR, Tompkins MB, Flowers WL, Whisnant CS, Almond GW. Effects of stress associated with weaning on the adaptive immune system in pigs. J Anim Sci. 2012;90: 649–656. 10.2527/jas.2010-3470 21926316

[39] Ramos-Romero S, Pérez-Cano FJ, Castellote C, Castell M, Franch A. Effect of cocoa-enriched diets on lymphocytes involved in adjuvant arthritis in rats. Br J Nutr. 2012;107: 378–387. 10.1017/S0007114511003035 21762542

[40] Salvatore S, Vandenplas Y. Prebiotics and probiotics in therapy and prevention of gastrointestinal diseases in children In: Watson RR, Preedy VR, editors. Bioactive Foods in Promoting Health. Elsevier; 2010 pp. 181–203. 10.1016/B978-0-12-374938-3.00013-X

[41] Veereman-Wauters G, Staelens S, Van de Broek H, Plaskie K, Wesling F, Roger LC, et al Physiological and bifidogenic effects of prebiotic supplements in infant formulae. J Pediatr Gastroenterol Nutr. 2011;52: 763–771. 10.1097/MPG.0b013e3182139f39 21593649

[42] Westerbeek E, Hensgens R, Mihatsch W, Boehm G, Lafeber H, van Elburg R. The effect of neutral and acidic oligosaccharides on stool viscosity, stool frequency and stool pH in preterm infants. Acta Paediatr. 2011;100: 1426–1431. 10.1111/j.1651-2227.2011.02295.x 21449921

[43] Gebert S, Davis E, Rehberger T, Maxwell CV. Lactobacillus brevis strain 1E1 administered to piglets through milk supplementation prior to weaning maintains intestinal integrity after the weaning event. Benef Microbes. 2011;2: 35–45. 10.3920/BM2010.0043 21831788

[44] Borruel N. Increased mucosal tumour necrosis factor alpha production in Crohn’s disease can be downregulated ex vivo by probiotic bacteria. Gut. 2002;51: 659–664. 10.1136/gut.51.5.659 12377803 PMC1773447

[45] Carol M, Borruel N, Antolin M, Llopis M, Casellas F, Guarner F, et al Modulation of apoptosis in intestinal lymphocytes by a probiotic bacteria in Crohn’s disease. J Leukoc Biol. 2006;79: 917–922. 10.1189/jlb.0405188.1 16641137

[46] Hu G, Yang S, Hu W, Wen Z, He D, Zeng L, et al Effect of cold stress on immunity in rats. Exp Ther Med. 2016;11: 33–42. 10.3892/etm.2015.2854 26889214 PMC4726882

[47] Castillo NA, Perdigon G, de Moreno de Leblanc A. Oral administration of a probiotic Lactobacillus modulates cytokine production and TLR expression improving the immune response against Salmonella enterica serovar Typhimurium infection in mice. BMC Microbiol. 2011;11: 177 10.1186/1471-2180-11-177 21813005 PMC3173335

[48] Amit-Romach E, Uni Z, Reifen R. Multistep mechanism of probiotic bacterium, the effect on innate immune system. Mol Nutr Food Res. 2010;54: 277–284. 10.1002/mnfr.200800591 19998380

[49] Liu Y, Fatheree NY, Mangalat N, Rhoads JM. Lactobacillus reuteri strains reduce incidence and severity of experimental necrotizing enterocolitis via modulation of TLR4 and NF-kB signaling in the intestine. AJP Gastrointest Liver Physiol. 2012;302: G608–G617. 10.1152/ajpgi.00266.2011 22207578 PMC3311308

[50] Hong M, Kim SW, Han SH, Kim DJ, Suk KT, Kim YS, et al Probiotics (Lactobacillus rhamnosus R0011 and acidophilus R0052) reduce the expression of toll-like receptor 4 in mice with alcoholic liver disease. PLoS One. 2015;10: 1–17. 10.1371/journal.pone.0117451 25692549 PMC4333821

[51] Yang X, Fu Y, Liu J, Ren H. Impact of probiotics on Toll-like receptor 4 expression in an experimental model of ulcerative colitis. J Huazhong Univ Sci Technol Med Sci. 2013;33: 661–665. 10.1007/s11596-013-1177-9 24142717

[52] Agace WW. Generation of gut-homing T cells and their localization to the small intestinal mucosa. Immunol Lett. 2010;128: 21–23. 10.1016/j.imlet.2009.09.012 19808049

[53] Ivetic A. Signals regulating L-selectin-dependent leucocyte adhesion and transmigration. Int J Biochem Cell Biol. 2013;45: 550–555. 10.1016/j.biocel.2012.12.023 23299028

[54] Cepek KL, Shaw SK, Parker CM, Russell GJ, Morrow JS, Rimm DL, et al Adhesion between epithelial cells and T lymphocytes mediated by E-cadherin and the a E b 7 integrin. Nature. 1994;372: 190–193. 10.1038/372190a0 7969453

[55] Kilshaw PJ. Alpha E beta 7. Mol Pathol. 1999;52: 203–207. 10.1136/mp.52.4.203 10694940 PMC395700

[56] Cauley LS, Lefrançois L. Guarding the perimeter: protection of the mucosa by tissue-resident memory T cells. Mucosal Immunol. 2013;6: 14–23. 10.1038/mi.2012.96 23131785 PMC4034055

[57] Chu ZX, Chen HQ, Ma YL, Zhou YK, Zhang M, Zhang P, et al Lactobacillus plantarum prevents the upregulation of adhesion molecule expression in an experimental colitis model. Dig Dis Sci. 2010;55: 2505–2513. 10.1007/s10620-009-1063-2 19960256

[58] Bernardo D, Sánchez B, Al-Hassi HO, Mann ER, Urdaci MC, Knight SC, et al Microbiota/host crosstalk biomarkers: Regulatory response of human intestinal dendritic cells exposed to Lactobacillus extracellular encrypted peptide. PLoS One. 2012;7: 1–8. 10.1371/journal.pone.0036262 22606249 PMC3351486

[59] Knight SC. Dendritic cell-T-cell circuitry in health and changes in inflammatory bowel disease and its treatment. Dig Dis. 2016;34: 51–57. 10.1159/000442926 26982806 PMC5022659

[60] Mann ER, You J, Horneffer-Van Der Sluis V, Bernardo D, Omar Al-Hassi H, Landy J, et al Dysregulated circulating dendritic cell function in ulcerative colitis is partially restored by probiotic strain lactobacillus casei shirota. Mediators Inflamm. 2013;2013: 573576 10.1155/2013/573576 23970814 PMC3732609

[61] Majamaa H, Isolauri E. Probiotics: a novel approach in the management of food allergy. J Allergy Clin Immunol. 1997;99: 179–185. 10.1034/j.1398-9995.1999.00103.x 9042042

[62] Park JH, Um JI, Lee BJ, Goh JS, Park SY, Kim WS, et al Encapsulated Bifidobacterium bifidum potentiates intestinal IgA production. Cell Immunol. 2002;219: 22–27. 10.1016/S0008-8749(02)00579-8 12473264

[63] Fukushima Y, Kawata Y, Hara H, Terada A, Mitsuoka T. Effect of a probiotic formula on intestinal immunoglobulin A production in healthy children. Int J Food Microbiol. 1998;42: 39–44. 10.1016/S0168-1605(98)00056-7 9706796

