# Supplementary material for: Preclinical Immunomodulation by the Probiotic Bifidobacterium breve M-16V in Early Life
Source: PLoS One. 2016 Nov 7;11(11):e0166082. doi: 10.1371/journal.pone.0166082 (PMC5098803; doi:10.1371/journal.pone.0166082)
Supplement: S1 Fig — A) In Panel 1, B cells (CD45RA+) were identified. The frequency of TLR4+ cells in the B cell subset was investigated, as well as in CD8+CD4- and CD8-CD4+ subsets. TLR4+ cells were also quantified in the total gated lymphocytes (not shown). B) In Panel 2, the αE integrin/CD62L pattern was studied in each of the subsets defined by the combination of the markers CD4 and CD8. C) In Panel 3, it could be differentiated NK (NK+TCRαβ-) cells from NKT cells (NK+TCRαβ+), and also NK-TCRαβ+ cells, which in combination with TCRγδ+ cells (obtained from the Panel 4) constituted the total of T cells. Moreover, the CD8-CD4+ cells in the NK-TCRαβ+ subset could be considered as Th cells, and the CD8+CD4- cells in this subset plus the TCRγδ+ cells (Panel 4) could be considered as Tc cells. The proportion of CD8+ cells in the NK+TCRαβ- subset (NK cells) was also studied. D) In Panel 4, the proportion of CD8αα and CD8αβ cells were studied in the CD8+ cells subset and also in the TCRγδ+ cells subset. (DOCX) [file pone.0166082.s001.docx]

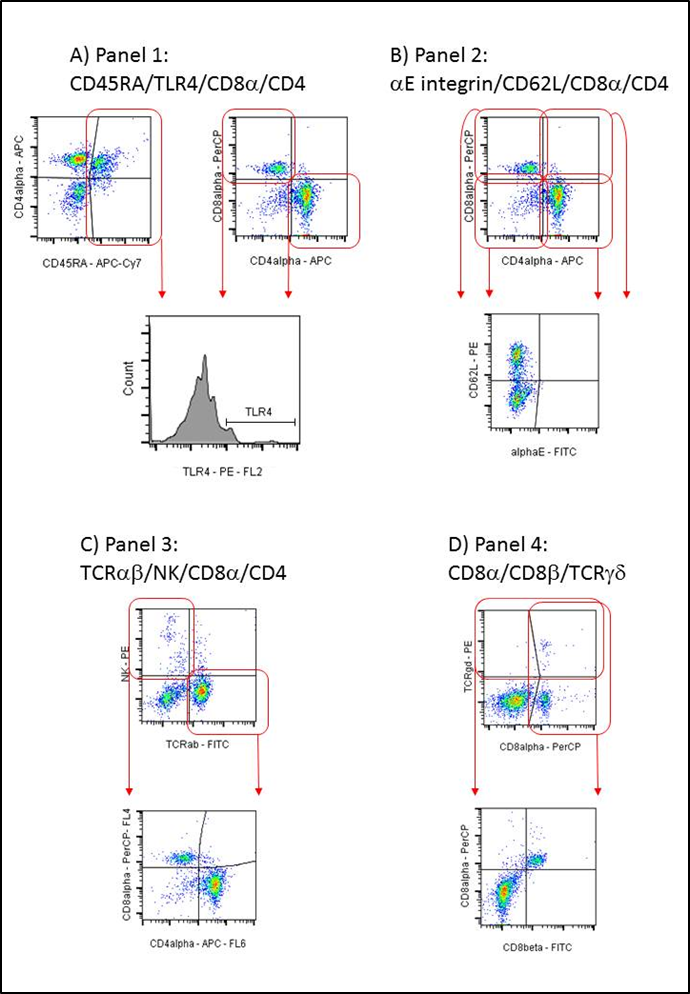


**S1 Fig. Example of the gating strategy using a mesenteric lymph node lymphocytes’ sample. A)** In Panel 1, B cells (CD45RA+) were identified. The frequency of TLR4+ cells in the B cell subset was investigated, as well as in CD8+CD4- and CD8-CD4+ subsets. TLR4+ cells were also quantified in the total gated lymphocytes (not shown). **B)** In Panel 2, the αE integrin/CD62L pattern was studied in each of the subsets defined by the combination of the markers CD4 and CD8. **C)** In Panel 3, it could be differentiated NK (NK+TCRαβ-) cells from NKT cells (NK+TCRαβ+), and also NK-TCRαβ+ cells, which in combination with TCRγδ+ cells (obtained from the Panel 4) constituted the total of T cells. Moreover, the CD8-CD4+ cells in the NK-TCRαβ+ subset could be considered as Th cells, and the CD8+CD4- cells in this subset plus the TCRγδ+ cells (Panel 4) could be considered as Tc cells. The proportion of CD8+ cells in the NK+TCRαβ- subset (NK cells) was also studied. **D)** In Panel 4, the proportion of CD8αα and CD8αβ cells were studied in the CD8+ cells subset and also in the TCRγδ+ cells subset.
